# Congenital Syphilis Coinfection in a Preterm Infant with Early Onset Sepsis due to *Enterobacter cloacae*

**DOI:** 10.1155/2021/1334846

**Published:** 2021-07-10

**Authors:** Sakviseth Bin, Sethikar Im

**Affiliations:** ^1^Neonatal Intensive Care Unit of Calmette Hospital, Phnom Penh, Cambodia; ^2^Department of Pediatrics, University of Health Sciences, Phnom Penh, Cambodia

## Abstract

**Introduction:**

Syphilis is a tropical disease, caused by a spirochete *Treponema pallidum*, which can be transmitted transplacentally from untreated mothers to the fetus during any stages of pregnancy. Clinical manifestations of early congenital syphilis are variable and nonspecific. The diagnosis is based on the serology status of the mother, newborn clinical symptoms, and comparative serology titer between mother and newborn. *Case Presentation*. A late preterm female infant, appropriate for gestational age, was treated for severe early onset sepsis due to *Enterobacter cloacae* since day 2 of life. The coinfection with *Treponema pallidum* was suspected and confirmed at day 4 with clinical signs and a fourfold increase of rapid plasma reagin (RPR) compared to mother's serology. Combined with meropenem and amikacin, Benzyl-Penicillin was used for 10 days, thereby resulting in a significant clinical and laboratory improvement. The girl was discharged at day 18 and brought for regular follow-ups for both growth milestone and syphilis serology. RPR decreased fourfold at the age of 1 month.

**Conclusion:**

Syphilis should not be overlooked. The vertical transmission is preventable by an on-time treatment of the infected mother, triggered by a proper antenatal screening at the right time. Congenital syphilis should be ruled out in any challenging neonatal sepsis. The diagnosis tools and treatments are easily accessible and inexpensive in our economical settings.

## 1. Introduction

Congenital syphilis results from the transmission of the spirochetes *Treponema pallidum* from an infected mother, untreated or inadequately treated, to the fetus mostly via placenta during pregnancy [1]. In Southeast Asia, the rate of congenital syphilis was 145 per 100,000 live births in 2016 [2]. Untreated maternal syphilis is associated with several adverse birth outcomes such as fetal loss, stillbirth, neonatal death, symptomatic infected newborns, premature delivery, and low birthweight [3].

Most live-born neonates with congenital syphilis are asymptomatic at birth [4, 5]. Furthermore, the presence of signs at birth reflects the timing of in utero infection and the timing of maternal treatment [6]. Early congenital syphilis is defined by the clinical onset before the age of 2 years. Clinical and laboratory manifestations are linked to the inflammatory responses of hosts during the active infection. However, they are variable and nonspecific. The most common symptoms include hepatomegaly, splenomegaly, jaundice, and skin rashes. Skin involvements are heterogeneous: petechiae and purpura due to frequent thrombocytopenia, unspecified copper-colored, desquamative maculopapular rash in the palms and soles, and rarely, the characteristic bullous lesions “pemphigus syphiliticus.” Skeletal and central nervous system (CNS) involvements are also common. Other symptoms, namely, lymphadenopathies, rhinitis, anemia, pneumonia, myocarditis, or even diarrhea, were reported [7].

The definitive diagnosis of congenital syphilis is established by the detection of the spirochetes by dark-field microscopy and direct fluorescent antibody (DFA) testing. Both methods, however, are not practically used as they require special equipment and experienced laboratory technicians. Therefore, the diagnosis and treatment plan rely on multiple factors such as maternal serology and treatment history, newborn physical examination, and quantitative neonatal serologic titers, paired with mother's, by using the same test and the same laboratory [8]. The nontreponemal tests, including RPR (rapid plasma reagin) and VDRL (Venereal Disease Research Laboratory), are the cornerstones of the diagnosis in newborn because of their simplicity, rapidity, and affordability. They provide a semiquantitative result, which can be used for the diagnosis by pairing with mother's titer, for monitoring treatment response, and for the follow-ups [9].

The drug of choice for congenital syphilis is parenteral penicillin G, the only proven antibiotic with effective spirochetocidal activities and the minimal toxicity. In case of allergy or shortage of penicillin, ceftriaxone can be used in newborn with careful clinical and serologic follow-ups [8, 9].

In this case study, we report a case of congenital syphilis, of which the diagnosis was delayed because the antenatal screening was negative, and *Enterobacter cloacae* was identified in hemoculture.

## 2. Case Presentation

The baby girl was delivered at 34 weeks of gestational age (WGA) by vaginal delivery. The amniotic fluid was clear, and the Apgar scores were 8 and 9 at M1 and M5, respectively. The birthweight was 2100 g (50^th^ percentile).

Her mother, 35-year-old, gravida 2 para 1 (G2P1), had no particular history and no pregnancy-related complications. The antenatal care was performed regularly in a private hospital; HIV and syphilis tests performed in the first semester were negative. At admission, she was afebrile, but her laboratory tests showed increased C-reactive protein (CRP) to 60 mg/L, complete blood count (CBC) within normal range, urine examination normal, and hemoculture sterile.

The newborn was admitted to neonatal intensive care unit (NICU) for respiratory distress and suspected neonatal sepsis. At admission, she was active and pink, with good muscle tone, but she had mild respiratory distress (Silverman score 2), enlarged liver (4 cm below the costal margin), and enlarged spleen (2 cm below the costal margin). No skin lesions were noted. She was put on nasal cannula with oxygen 2 L/min. As per unit protocol, empiric antibiotic therapy with cefotaxime, ampicillin, and amikacin was given. The chest X-ray showed a typical transient tachypnea of newborn.

At day 2 of life, the peripheral blood culture that had been obtained at admission grew Gram-negative bacillus. The investigation showed severe thrombocytopenia (platelets 12 Giga/L, in Table 1) and increased CRP (33.9 mg/L). Due to thrombocytopenia, we did not perform lumbar puncture (LP) to rule out meningitis. Thus, we switched to meropenem 20 mg/kg/dose every 8 hours and amikacin 10 mg/kg/day.

At day 4 of life, *Enterobacter cloacae* was identified with confirmed sensitivity to meropenem and amikacin (Table 2) but clinically not improved. With noted hepatosplenomegaly and worsening inflammatory markers (CRP 122.9 mg/L), we requested the syphilis test for the newborn and the mother, which later confirmed the diagnosis of congenital syphilis, with infant's RPR 1 : 32 (fourfold the mother's RPR 1 : 8, given in Table 3).

We defined and managed our case in accordance with the US Centers for Disease Control (CDC) and Prevention's guidelines [8]. Our case met 2 of the 3 criteria of “proven or highly probable disease:”

1. An abnormal physical examination that is consistent with congenital syphilis
2. A serum VDRL or RPR titer that is ≥fourfold the corresponding maternal titer
3. A positive dark field or fluorescent antibody test of body fluid (s), placenta, or umbilical cord

Therefore, we started the CDC recommended ten-day regimen of Benzyl-Penicillin® (0.96 g/1.6 Million IU) at day 4 of life with 50,000 units/kg/dose IV every 12 hours for 7 days and then every 8 hours for 3 days. The infant was closely observed during the first 24 hours after administration; there was neither anaphylactic nor Jarish–Herxheimer reaction reported. The babygram was normal, and the ultrasound of the brain and the heart were within the normal range.

The premature girl responded well to the treatment. Feeding difficulties were resolved; she was weaned off oxygen at day 9; hepatomegaly decreased to 2 cm below costal margin and spleen not palpable. CRP decreased to 12.9 mg/L. Platelets increased progressively to 94 Giga/L at day 16 after 2 platelet transfusions. Finally, she was discharged at day 18 of life with corrected age of 36 WGA and 4 days.

She was brought for regular follow-up to evaluate growth and possible neurological complications. During her stay, she had cholestatic jaundice and the abdomen ultrasound ruled out biliary atresia. She was treated with ursodeoxycholic acid 20 mg/kg/day in 2 doses, with good response.

For serological follow-up, the infant's RPR titer decreased fourfold since the age of 1 month and remained at 1 : 8 at 3 months (Table 3). The girl will be followed-up every 3 months until the age of 1 year.

## 3. Discussion

The estimated global rate for congenital syphilis declined from 540 in 2012 to 473 per 100,000 live births in 2016. However, congenital syphilis remains a global burden since the prevalence of maternal syphilis worldwide remains stagnant, with 0.69% in 2012 and 0.70% in 2016 (690 per 100,000 women) [2].

A systematic review conducted by Gomez et al. showed the rate of premature delivery about 5.8% and clinical evidence of congenital syphilis about 15%, which are higher in untreated syphilis mothers than in pregnant women without syphilis [3].

Approximately 80% of neonates with early congenital syphilis are asymptomatic [4], and the most common clinical findings include hepatosplenomegaly, jaundice due to hepatitis or cholestasis, rash, rhinitis, lymphadenopathies, and skeletal involvement. Hepatomegaly occurs in most symptomatic infants (71%), associated or not with splenomegaly (60%). Hematologic abnormalities may include anemia, thrombocytopenia, and leukopenia or leukocytosis [6, 10]. In symptomatic cases, bone lesions (most commonly osteochondritis and periostitis) occur in about 60% and neurosyphilis about 50% [7].

In our case, the infant was born prematurely at 34 WGA with clinical symptoms. Organomegaly was remarkable since admission; however, we did not initially consider congenital syphilis as the routine maternal screening was negative, and it was not retested near the delivery time. Moreover, the diagnosis was masked by confirmed sepsis due to *Enterobacter cloacae*. The diagnosis was done at day 4, and we noted a remarkable clinical and serological response to the 10-day regimen of Benzyl-Penicillin. There were neither skeletal nor CNS involvements. RPR decreased fourfold early since the age of 1 month.

There are some limitations of our report. First, the recovery might not be completely due to Benzyl-Penicillin as the infant was also treated simultaneously with meropenem for 14 days to treat *Enterobacter cloacae* (until 7 days after hemoculture negative). Second, due to severe thrombocytopenia, we did not perform LP early to rule out CNS invasion. We used high dose of meropenem, and LP done at day 17 was normal. During the follow-ups, as neurological assessment and growth chart were normal for age, we did not repeat LP.

## 4. Conclusion

The mother-to-child transmission of syphilis is preventable with proper antenatal screenings and adequate on-time maternal treatment. However, the early diagnosis of congenital syphilis is quite challenging as it is mostly overlooked due to a negative antenatal screening or other infectious diseases in NICU. Our case report suggests that pregnant women should be retested for syphilis since it can occur in later stages of pregnancy, and congenital syphilis should be ruled out in any severe challenging infectious cases.

## Figures and Tables

**Table 1:** Laboratory investigations during hospitalization.

	Day 2 of life	Day 4 of life (PNC G D1)	Day 9 of life (PNC G D6)	Day 16 of life	Follow-up (1 month)
White blood cell (4-9 Giga/L)	10.9	17.5	17.8	14.7	12.7
Hemoglobin (13–17 g/dL)	12.8	12.1	12.8	18.9	15.5
Platelets (150–450 Giga/L)	12	12	21	94	135
C-reactive protein (<5 mg/L)	33.9	122.9	12.9	8.14	—
Procalcitonin (<0.5 ng/ml)	—	9.45	0.28	0.421	—
Hemoculture	*Enterobacter cloacae*	—	Negative	Negative	—

**Table 2:** Antibiogram of *Enterobacter cloacae*.

Specimen	Blood
Gram stain	Gram-negative bacilli
Culture and identification	*Enterobacter cloacae*
Sensitivity	
Ampicillin	R
Amoxiclav	R
Cefazolin	R
Ceftriaxone	Sensible
Ceftazidime	R
Imipenem	R
Meropenem	Sensible
Ertapenem	Sensible
Gentamicin	Sensible
Amikacin	Sensible
Co-trimox	Sensible
Nalidixic acid	I
Ofloxacin	Sensible
Ciprofloxacin	R
Fosfomycin	Sensible
Piper/tazobactam	Sensible
Cefepime	Sensible

**Table 3:** Syphilis serological follow-up

	Mother	Newborn		
	Jan 27	Jan 27 (day 4 of life)	1 month	3 months
RPR	1 : 8	1 : 32	1 : 8	1 : 8
TPHA	1 : 320	1 : 640	1 : 1280	1 : 320

## Data Availability

The data used to support the findings of this study are available from the corresponding author upon request.
